# Visual Algorithm of VR E-Sports for Online Health Care

**DOI:** 10.3390/healthcare9070824

**Published:** 2021-06-29

**Authors:** Sang-Guk Lim, Se-Hoon Jung, Jun-Ho Huh

**Affiliations:** 1 School of Culture Contents, Youngsan University, Busan 48015, Korea; gooki7@ysu.ac.kr; 2 School of Creative Convergence, Andong National University, Andong 36729, Korea; 3 Department of Data Science, (National) Korea Maritime and Ocean University, Busan 49112, Korea

**Keywords:** VR, virtual reality, VR e-sports, AI, immersive content, visual algorithm, visual system, online health care, game

## Abstract

The need for non-face-to-face online health care has emerged through the era of “untact”. However, there is a lack of standardization work and research cases on the exercise effect of immersive content. In this study, the possibility of the exercise effect of VR e-sports among e-sports cases were presented through a visual algorithm analysis. In addition, the evaluation criteria were established. The research method compares and analyzes e-sports cases and VR e-sports cases by applying existing evaluation research cases. It also sets up a new evaluation standard. As for the analysis result, the device immersion method and interaction range were set through an algorithm analysis; FOV and frame immersion were set through typification; the user recognition method and interaction method were set through the visual diagram. Then, each derived result value was quantified and a new evaluation criterion was proposed.

## 1. Introduction

The COVID-19 pandemic has not only brought confusion and control to reciprocal exchanges, as well as the entire society and economy, but also is causing mentally and physically serious illnesses. In particular, one of the causes is the absence of exercise, along with limited physical activities. With the prolongation of the pandemic, however, these have increasingly emerged as serious issues, whose resolution will require different non-contact approaches to activity and alternative environments [1]. A new coinage, “ontact,” was made amidst these changes [2]. It is a concept of adding “on,” which represents connecting to the outside world online, to “untact,” which means non-contact. That is, it is an online in-person approach, emerging as a new flow, following the extended spread of the COVID-19 pandemic in 2020. Living in an era when people live to be 100 years old, modern people concern themselves with health and make efforts to keep their health in their busy daily life. Health care has now become an essential element in such a life [3]. After the outbreak of COVID-19, our daily lives have halted, and our physical activities restricted. In addition, functional restoration of the body for respiratory improvement after eradication of the virus is important, and in that respect, rehabilitation plays an important role in acute COVID-19 management [4]. As a way to overcome this crisis, interest in personal health care-type immersive contents based on physical exercise is increasing. Games or e-sports [5] are essential to the culture of indulging in leisure and pleasure, even briefly, to keep physical health and release mental stress. In recent years, e-sports have settled down as part of play culture among most of the young generation and formed a global fandom culture [6].

E-sports is a method of online communication over the Internet through screen devices, generally in the form of games. Recently, it has also established itself as a sport through the competitive structure, and it can be expanded from a culture of enjoying alone to a “health care”-type sport that experiences and communicates through an online network and achieves goals based on physical exercise.

Therefore health care-type immersive content has huge expandability as indoor sports fit for the “ontact” trend amidst the COVID-19 pandemic. There are various types of games according to devices, including mobile games, representative RPG games on a PC, home console games, and VR games. VR e-sports as an extended concept are part of the newly emerging extended reality (XR) culture.

In other words, VR e-sports are not only a fun game, but also has a higher screen scalability than existing e-sports and an excellent sense of immersion through interaction with the human body and the device. In addition, VR e-sports, which have been expanded to AR and MR, have sufficient potential for development as personal health care-type immersive content based on physical exercise. Therefore, it seems that VR game-type health care that is revitalized to meet the needs of the times is necessary. However, most of the currently distributed health care products are monitor-based, one-way products, and the standard for the exercise effect has not been established. In particular, from a technical point of view, it is a method of following the actions shown through a small frame monitor and checking the user’s actions using sense. Most of them are focused on the operation method rather than the fun, so we think the immersion is also significantly lower. From an academic point of view, there are no academic research results or empirical evaluation standards for its effectiveness, and it is true that public awareness of how to use it is still lacking. In addition, in the case of virtual reality devices that have been continuously developed in recent years, the standardization process for the resulting values has not been established, and the operating system through “VISION” has not been clearly established.

Recently, the importance of physical activity has been highlighted in preparation for the post-COVID-19 era. Therefore, it suggests the possibility of safe, diverse and fun online e-sports health care in terms of prevention and rehabilitation. In the process, we intend to expand the fun elements of e-sports and the possibility of substituting physical activity through VR e-sports. In other words, by proposing a new evaluation tool that can measure the impact of VR e-sports on physical activity as a role of prevention and rehabilitation during a specific pandemic, the direction of realistic health care to be produced in the future is presented and the evaluation necessary to verify its effectiveness. The purpose of this study is to present a tool.

Findings about various forms of health care-type immersive content have been recently published overseas in the fields of medicine and fitness, for example the “Superpower Glass Intervention” project based on Google Glass, whose VR-based effects were tested for the behavioral analysis and treatment of autistic children [7]. A case study applied VR to the treatment of lumbar pain [8]. In another case, VR gaming technologies were applied and proven effective for the relief of pain in physical therapy [9]. Additionally, a study case on the rehabilitation efficacy of VR through comparison of virtual reality rehabilitation and conventional rehabilitation in Parkinson’s disease [10].

In addition, studies on osteoporosis in patients with Down’s syndrome [11] and studies on various health-related side effects caused by sarcopenia [12] remind us once again how important health and rehabilitation are in our lives.

A Korean smart health care company, Omni C&S, incorporated VR technologies into its smart health care solution OMNIFITMindcare, which helps to manage mental health [13]. Another South Korean company, Kakao VX, made a home exercise equipment called “VR Smart Home Training” and developed a variety of content, including OhShape [14]. Samsung announced a VR health care solution to help users exercise at home by themselves by connecting their avatars to artificial intelligence. Health-related research and devices are launched in various forms.

These are, however, at the experimentation stage. Research is being conducted on clear standardized methods to measure exercise or its effects with a lack of public awareness of them. Basically, how do you get users to engage? Under what conditions can exercise continue? How to build an evaluation of the effectiveness of home health care without environmental risk factors? etc., are presented as research questions. In this study, we intend to propose a new evaluation method to verify the experience method of general health care products. In addition, there is a need for methodology to examine their sustainability, since they are for health care practices by oneself at home. The present study, thus, proposed an analysis framework to test exercise effects, based on a methodology of applying and keeping gaming methods to such health care-type immersive content through the comparison analysis of e-sports and VR e-sports, as well as the result values of visualization of algorithms established in the research process. The findings of the study may serve as a guide for developers to apply to the UI/UX of immersive devices and as a humanistic guidebook for users to understand a method of experiencing and enjoying extended reality. The investigator employed such research methods as examining various research cases and visualizing a cognitive system built by immersive devices and physical activities in an algorithm form. Results values were used to propose an analysis framework to test exercise effects. An experiment was conducted to quantify differences in physical activities between the game rules of e-sports and those of VR e-sports, and to examine their efficiency as exercise effects through qualitative assessment.

After visualizing an operating algorithm between an immersive device and its user, the investigator used the result values as an analysis framework to compare and assess e-sports and VR e-sports in exercise effects. By introducing “ontact” cases in preparation of the post-COVID-19 era, the present study proposed a way of managing personal health, as well as finding joy in daily life. It will serve as a safety net to maintain our daily life in a never-ending war against another virus.

## 2. Theoretical Background

### 2.1. Case Study

Recently, many research cases related to health care have been published due to COVID-19 and are being commercialized. Most products deliver information visually through a 2D-based monitor. It is a way to compete and immerse yourself by recognizing the user’s motion and interacting with the character in the screen to verify and score the exercise effect. In this context, the operating method of e-sports is very similar. Sit at a table and use a 2D monitor to control and immerse in the movement of the character you control and compete with your opponent online. The interest in e-sports is evident from the recent various humanistic research cases, and academic research efforts are emerging as well as technological developments. Given the periodic characteristics of today in a mix of virtualism and reality, there is a need to investigate the structural understanding and visual perception process of “vision” or looking at an object. The meanings of vision are expanding with mobility added to the basic screen forms, including VR, AR, MR, and physical computing in a wearable [15] method, which allows one to wear a device on the body and secure a direct view. New spaces of vision mediated in this way are post-Cartesian, post-perspective, and post-physical, but still remain within the limits of frames on a screen [16]. Visual illusionism represents the history of reproduction around presence from Giotto, before the law of perspective, to da Vinci that began to use the law of perspective in full scale [17]. Making an algorithm for the stages of visual information processing, including sense, perception, and cognition, in a visual system fit for the digital era involves the ability [18] to recognize and distinguish visual stimuli and understanding stimuli from the connection of previous experiences and perceptions, rather than responding to various human senses. It is also to sublimate it as a role and value of culture, through the academic interpretation of technological development.

Vision requests the interpretation and insight of a subject that is “cognitive,” rather than a simple physical act of “seeing” by the dictionary definition [19]. When an observer sees an object through his or her eyes, it is necessary to obtain information from the visual characteristics of the object, categorize it, classify it, and select it. [20]. The figure illustrates that the human visual structure is recognized through various devices and leads to physical activities. Analyzing such a process and turning it into an algorithm through e-sports are required in the development of various devices, in addition to immersive content. As a recent study on e-sports, there have been studies on e-sports user behavior and development of e-sports measures [21]. There was various research, including the one [22] on the effects of physical activities in virtual reality, in “Experience on Demand” by Jeremy Bailenson. Health care-related research on neurological disorders and strokes examined cases of overcoming lumbar pain through virtual reality and reported that it reduced pain [23]. Another research used VR therapy to treat arachnophobia. Hoffman and Peterson published a research paper in the medical journal, PAIN in 2000, reporting that virtual reality reduced pain more than general games with the distraction technique in “Spider World” [24]. Other researchers analyzed the visual system. Hal Foster (2012) presented his study on the modern visual system in his “Vision and Visuality” [25]. Jeong Jeong-ju (2014) published a “Study on the Expansion of Communication in Media Art with the Window Metaphor” [26]. Lim Sang Guk and Kim Chee Yong (2018) proposed a digital visual system fit for the 21st century in their “Study on Changes to Digital Visuality in the 21st Century,” based on Lacan’s “notion of the real gaze” [27]. Still others conducted research on algorithms related to the visual system. In his “Study on the Visualization Methods of Poetry with Algorithm-Based Modeling,” Kim Ju-seop (2013) used “poetry” to turn images into algorithms [28]. Kim Min-seok, Choi Woo-seong, and Jeong Sun-yeong (2018) published “Design and Implementation of an Algorithm Visualization-Based Cluster Analysis Learning System” [29]. Lim Sang Guk (2020) built “An HMB-Based Interactive Immersive Media Algorithm with L-System” [30].

Developed by Aristid Lindenmayer in 1968, the parallel rewriting system, “L-System” was introduced into computer graphics by Alvy Ray Smith in 1984. Today, it serves useful purposes in the procedural modeling of plant growth, among other things [31]. Using L-System, Kim Ju-seop (2013) proposed a method of recreating each poem in the form of an organic tree in nature, reflecting their unique characteristics in the digital space by limiting the text scope to the literary genre of “poetry,” using algorithm-based modeling (procedural modeling). Kim Ju-seop (2013) offered a special explanation that L-System consisted of symbols and rules that replace symbols. Park Jin-wan visualized and presented Korean genealogy in “Visual Genealogy” to create a new story, rather than functionality or aesthetics [32]. Lim Sang Guk (2020) visualized cases of immersive content devices recently used across various fields through the analysis of their visual systems based on image categorization and text listing. He was able to understand the characteristics of media and methods of seeing by the period and offer a guide for UI/UX analysis in media development, based on comparison results.

### 2.2. Study Case of Visual Algorithm Realization

**Figure 1:**
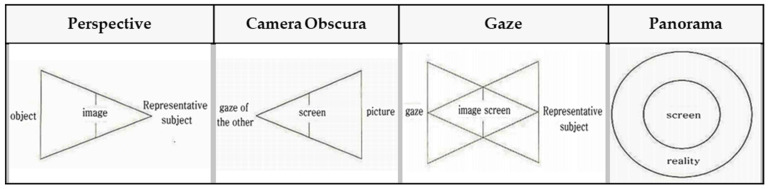
Lacan’s “notion of the real gaze”.

The investigator had to visualize or categorize a visual system to build a visual algorithm needed in the present study, and further, how to recognize and utilize visual information into images in the process of visual information processing, including sense, perception, and cognition in relations between diverse devices and users. Therefore, a method for algorithmizing the visual system was established, and the cases were investigated. To date, there is no evaluation tool that can analyze and verify the digital visual system. However, in this study, we would like to propose a tool for visual system analysis through various cases priority, this paper intends to utilize the following four types of representative papers. In one of such research cases, Jeong Jeong-ju (2014) proposed a traditional Cartesian visual system [33] that divided the traditional visual system into three types, including perspective, camera obscura, and panorama, in “Study on the Expansion of Communication in Media Art with the Window Metaphor.” In his “Vision and Visuality,” Hal Foster (2012) introduced a case of the notion of the real gaze for a visual system about Lacan’s “gaze” under the traditional Cartesian visual system. Crary insisted on a need for observers recognizing vision that had nothing to do with an “act of looking” [34]. Lacan maintained that the “Cartesian visual model” should inevitably be replaced with a new visual model capable of containing a sense mechanism via the nerves [35]. Based on these arguments, Lim Sang Guk (2017) in his “Study on the Characteristics of Visuality Changes and the Expansion of Digital Frames in the 21st Century,” in addition to Lim Sang Guk and Kim Chee Yong (2018) in their “Study on Changes to Digital Visuality in the 21st Century based on Lacan’s Notion of the Real Gaze,” reorganized the human visual system into a digital visual system of the 21st century and visualized it into a “notion of the real gaze” by using Lacan’s “notion of the real gaze”. An example of the research case of “Construction of HMD-based interactive immersive media algorithm using L-System” by Im Sang-guk (2020) is given. Among them, let us look at the research results of Im Sang-guk (2017), Im Sang-guk, and Kim Kim-yong (2018), which are research cases on changes in the visual system. The result is shown in Figure 1. As seen in Figure 1, the above-mentioned research cases defined the modern visual system of four elements, including perspective, camera obscura, gaze, and panorama, based on the “notion of the real gaze”.

**Figure 2:**
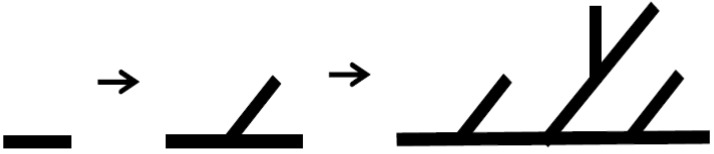
Example of branch generation using L-System.

In Figure 1, the notion of the real gaze is a visualized image of a dual visual system in a digital device. The “window” at the center is an “image screen” showing images. That is, screens in perspective are considered as “windows” to figure out an object by viewers as the origin of the term perspective, which means “seeing through” other spaces beyond the screen, suggests [36]. Viewers look at the apex (vanishing point) on the right through a window from the left side. However, in Lacan’s viewpoint, “gaze“ represents another eye to look at viewers. On the other hand, on a digital visual system, viewers look at moving images (character) on a liquid crystal display, rather than a vanishing point in the traditional perspective. Such images are virtual images reproduced by the computer. This relationship leads to the formation of interactions between viewers and devices. As a study related to text visualization of images, Kim Ju-seop (2013) reported that character strings in L-System worked as a series of orders to draw Figure 2, based on algorithms. That is, it is “F -> F [+F] F”. In character strings of current rules, all “F” symbols are replaced with “F [+F] F”. As this rule is applied twice, it expands into the next character string.

Following “F -> F [+F] F -> F [+F] F [+F [+F] F] F [+F] F,” geometric meanings were granted to each symbol:

- F: drawing ‘_’ clockwise at the current position;
- +: changing the direction at 45 degrees counterclockwise;
- [: saving the current position;
- ]: returning to the position saved last.

**Figure 3:**
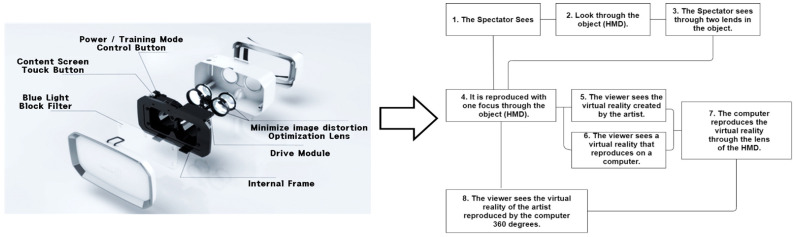
HMD visual system algorithm using L-System.

In this context, as if implementing an image through a string, we intend to apply the method of reconstructing text algorithmically through an image to this study. Figure 3 presents a case of applying users’ HMD-based cognitive process to L-System and turning the process into an algorithm in the rewriting method of character strings with a VR device in Lim Sang Guk (2020)’s “An HMB-Based Interactive Immersive Media Algorithm with L-System.” The process of users looking at an HMD device is sequenced in texts, and problems with the process, including dizziness and difficult cognition, are checked out in the regeneration process of images to figure out the process of viewers communicating with information images. As seen in these two research cases, cognition relations between devices and viewers were turned into a visual algorithm through the interpretation of images, based on a system of visualizing texts or rewriting character strings.

The next case categorized the visual systems of various immersive devices and visualized them through the “notion of the real gaze”. As seen in Table 1, recently launched immersive devices were listed according to the degree of immersion in the monitoring method of single frames generally used.

In the enjoyment of e-sports, the basic key is the duration of exercise to generate personal exercise ability effects. You need to exercise consistently for a long period in order to experience the effects. In this sense, immersion is an essential element in exercise effects. In relations between devices and users, a frame is a basic component of vision and important element to enhance immersion. The physical size of a frame works to increase immersion, expand the scope of vision, and maximize movement in the users’ enjoyment of e-sports. In other words, there are clear differences in effectiveness between single frames on the monitor screen for immersion, movement of gaze, and activity of the body, and 360-degree spaces for complete immersion and expandability of gaze.

A VR device, HMD, creates a 360-degree space by blocking a gaze in a complete real space. Although it excels in immersion, it can have limits in physical activities. Users have to enjoy a game on the original spot, since they have no visual field secured in a real space. Of device cases proposed as solutions, AR and MR have the greatest advantage of allowing users to enjoy virtual images together in a real space. The expandability of frames represents a physical field of view. As its scope expands, immersion enhances and exercise effects are maximized. In this context, the cases in Table 1 were examined to figure out changes to users’ visual frames. A field of view of approximately 90° is created for a single frame, and one of approximately 180~270° is created for an extended frame. Body movements are shown in a limited manner, according to the scope of field of view. VR, on the other hand, maximizes immersion with a full 360° space. Users are, however, restricted for their physical activities, due to the blockage of real spaces, which points to a disadvantage that they have to stand still or sit down to play a game. Unlike VR, AR allows users to play a game, while looking at a real space in a 360° space. In AR, exercise effects are maximized, as users are allowed to move their hands and move around easily. Furthermore, it can function as a media-based tool. In AR, however, augmented images are narrow within the limited media frame size of a field of view, thus lowering immersion. MR supplements the disadvantages of VR and AR and highlights their advantages. Users can secure a field of view of 360° and move their bodies freely, which suggests that MR can serve as a media-based exercise machine.

### 2.3. Interactive Visual Algorithm Visualization Research Case

**Figure 4:**
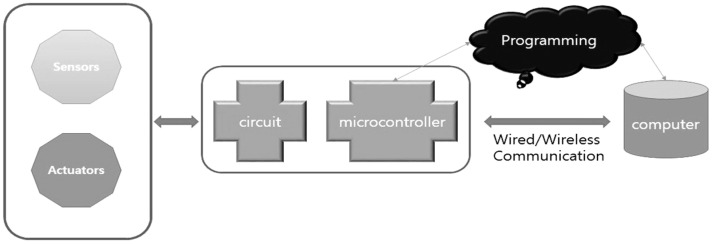
Principles of physical computing.

One of the characteristics of the vision perspective in the digital era of the 21st century is a dual visual system. As seen in Table 1 above, the human vision system moves toward a three-dimensional system beyond a two-dimensional one. In this digital convergence era, various realities coexist together including actual reality, virtual reality, and augmented reality. In other words, the recent converged media is characterized by mutual communication and interactions between users and their devices, through optical seek-through and display-based interactive methods. As illustrated in Figure 4, the most important part in this mutual interactive method is the perception of the body through physical computing or the kinetic and tracking principle. Following the development of media, viewers’ bodies have been a central research subject in various aspects. The keyword of viewer participation in works through the body is the biggest interest of contemporary artists and a characteristic of digital art in the field of digital art. The bodies of viewers represent their interactive natural ego in works and are considered as subjects of perception in the traditional concept of cognition. Now, people have to see and feel with their bodies in the sense of vision with the simple perspective of visual frames replaced by the body perception perspective of frames.

In his study “An HMB-Based Interactive Immersive Media Algorithm with L-System,” Lim Sang Guk (2020) classified interactive immersive media into three types in Table 2. They are “inter-media people,” “communion,” and “in-media people” types. In the first “inter-media people” type, viewers look at a monitor and act accordingly, and their acts are recognized through kinetic sensors and trigger the reactions of images on the screen. Viewers interact with the monitor through camera sensors detecting their physical movements, recognize it visually, and move along with it. In their movements, they form ties with characters on the screen and interact with them through their visual body cognition. In the next “communion” type, viewers touch the screen themselves. Unlike the objective “inter-media people” type, the “communion” type involves tactile interactions through direct body touches. Viewers touch the monitor screen directly and move accordingly, feeling reactions on the touch screen themselves. There are huge differences in the amount of exercise, according to frame sizes and degree of relationship with the screen. This type enhances viewers’ immersion further. In the last “in-media people” type, viewers interact with a work by moving their bodies in it. That is, they become a part of the work. The scope of frames builds a three-dimensional space, and viewers increase their activity level through their body movements and maximize their immersion.

These research cases show that the digital visual systems of the 21st century are based on moving images and viewers’ participation. With the involvement of a medium called the computer, based on interactions, an interactive dual visual system is built. Viewers identify with beings (characters) in the media by moving themselves at a position facing a work. Then, they develop their tactile sense by touching the media (touch screen), being divided into two subjects (viewers in reality and virtual viewers in the media) and becoming immersed at the boundary (screen). Lastly, they walk into a work and become the work (subject) itself. Table 3 shows outcomes of reproducing interactive case analysis results in Lacan’s visual plate.

These findings indicate that the scope of frames was limited in viewers’ relationships with a screen facing them, given the amount of exercise according to the fields of view, immersion, and activity scope of viewers facing a work in the “inter-media people” type. In the “in-media people type,” the amount of exercise increased, according to the expanding frames, widening fields of view, and rising utilization rate of spaces.

## 3. Analysis Method

### 3.1. Analysis Targets and Applicable Devices

The present study proposed and applied an analysis method based on these various research cases. It needed objects of analysis and devices to be applied to test the exercise effects of e-sports and VR e-sports, and to propose plans for their vitalization. In other words, the study needed to undergo a process of textualizing and visualizing connections in the cognition method of “vision-body” between various devices and users. This was followed by quantifying results values and testing through qualitative evaluation. Based on the research cases above, the study then proposed objects and frameworks of analysis. First, the objects of analysis in the study included three of the most popular games in the e-sports industry of South Korea and three of the most popular games in VR e-sports. As seen in Table 4, the top three popular games in e-sports in the nation were “League of Legends,” “Battleground,” and “StarCraft: Remastered.” The top three popular games in VR e-sports were “VR Beat Saber,” “VR Dragon Flight”, and the MR e-sports game, “HADO”.

The cases of e-sports in the nation are basically divided between mobile games, based on a smartphone, and RPG games, based on a PC. The present study focused on PC games with great frame expandability. The cases of VR e-sports selected in the study included the HMD-based VR game “Beat Saber,” the special force VR game “ Dragon Flight” version, and the MR-based game “HADO”.

Second, devices related to the objects of analysis were applied based on the cases of immersive devices in Table 5. That is, the characteristics of five representative immersive devices in their instructions were categorized in relations between users and their devices.

Third, relationships in the cognition method of “vision-body” between users and their devices in the game management method were applied to L-System, based on the objects of analysis and immersive devices to propose an analysis framework. This process was described in the rewriting method of character strings. Based on the algorithm analysis frameworks in Kim Ju-seop’s (2013) “Study on the Visualization Methods of Poetry through Algorithm-Based Modeling” and Lim Sang Guk’s (2020) “An HMB-Based Interactive Immersive Media Algorithm with L-System,” the visual cognition processes of objects of analysis, devices, and users were turned into algorithms through texts.

Fourth, the texts that were turned into algorithms were categorized into visual images and the “notion of the real gaze” based on the “notion of the real gaze” proposed in “Study on Changes to Digital Visuality in the 21st Century, based on Lacan’s Notion of the Real Gaze” of Lim Sang Guk and Kim Chee Yong (2018). Result values of fields of view were obtained to test immersion based on the scope of the image frames.

Fifth, the study digitized body movements seen through interactive relations between users and their devices and tested users’ exercise effects in their utilization of objects of analysis and devices. That is, it digitized the utilization scope of spaces, based on the movement degree and travel scope of users’ eyes, hands, feet, and bodies in the gaming method.

### 3.2. Proposed Experiment Method and Analysis Tool

**Figure 5:**
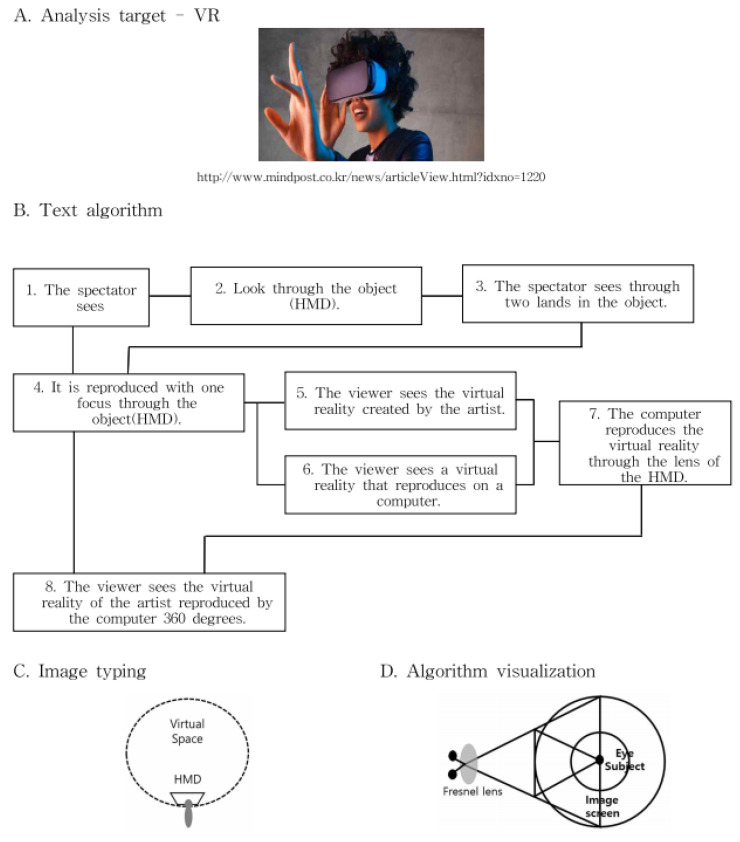
Experiment method model.

The study applied the analysis frameworks for the experimentation methods according to the analysis methods in Figure 5.

As seen in Table 5, the analysis framework used in the experimentation method underwent the process of A, B, C, and D, which involved selecting objects of analysis, applying them to L-System, and turning them into algorithms, based on texts in the rewriting method of character strings, categorizing them into images through algorithm analysis, and turning them into “notion of the real gaze”. This process generated the result values of user experiences and exercise effects in e-sports and VR e-sports.

Based on the analysis frameworks of A, B, C, and D above, the study proposed analysis criteria for result values to be generated. The criteria would cover result values under each analysis framework and tests of exercise immersion, scope, and effects in e-sports and VR e-sports. Under the framework of “A” for objects of analysis, killer content and devices were selected to be used in e-sports and VR e-sports, based on cases needed to measure fun and exercise effects for exercise persistence, as discussed above. The framework of “B” for the textualization of algorithms found rules of various devices in users’ cognition process and grounds for users’ immersion and fun, based on their characteristics. The framework of “C” for categorization expressed relationships between users and their devices in images and measured their immersion and scope of activities, based on fields of view through their frames. Users’ amounts of exercise were also measured, according to the scope of their space utilization and methods of content management, based on the categorization of characteristics of killer content and devices into images. The final framework of “D” for “notion of the real gaze” offered some grounds to predict UI/UX according to users’ immersion and interactive management with their devices, as well as the cognition methods of their bodies. Based on the outcomes, the study proposed a guide to predict killer content to be created and methods for users to utilize their devices. The analysis criteria can be found in Table 6.

Seeing is a process of an observer looking at an object with his or her eyes, obtaining information from its visual characteristics, categorizing and classifying it, and making a choice [37]. Wong (1994) reported that shapes, sizes, positions, and colors accounted for the most important parts in the visualization of conceptual elements through one’s eyes [38]. According to Stephen, sizes, shapes, spaces, and colors of visual elements are very important comparison elements [39]. In this context, it is possible to check which sensory organs are used by users in their devices for immersion under “B” of turning relationships between users and their devices into algorithms, based on texts, according to the criteria of experimental evaluation in Table 6. It is also possible to measure their scope of activities by tracing their body movements moving from the *x*-axis to the *y*-axis or traveling along the x-, y-, and z-axes in their interactions with their devices. Under “C,” it is possible to categorize instructions between users and their devices into visual images and visualize the degree of their frame utilization in checking their fields of view and uses of their devices. In VR HMD, a field of view (FOV) is important because it plays a big part in increasing the sense of reality in virtual reality. After the visual system of camera obscura, panoramas expanded the physical sensory experiences of subjects in realistic and verified spaces established by the law of principle based on the effects of technological reproduction [40].

Human eyeballs have an average of 110 FOV. An experiment can help to obtain FOV, secure FOV, and promote communion with a device to check user’s immersion. Under “D” of the “notion of the real gaze” between users and their devices, it is possible to visualize which process is used by users to communicate and commune with which sensory organs through which body parts. The result values can be used to predict the efficiency and measurement criteria of exercise effects. The evaluation criteria in Table 6 were used as analysis frameworks in the experiment of the study. Additionally, the scientific numerical range for measuring the effects of e-sports and VR e-sports exercise is defined as shown in Table 7.

### 3.3. Experiment Method

In the experiment, an experiment model was built based on these analysis frameworks and applied to the e-sports and VR e-sports to be analyzed. A database was built with the result values to propose an automation system to apply various devices and provide their result values.

**Figure 6:**
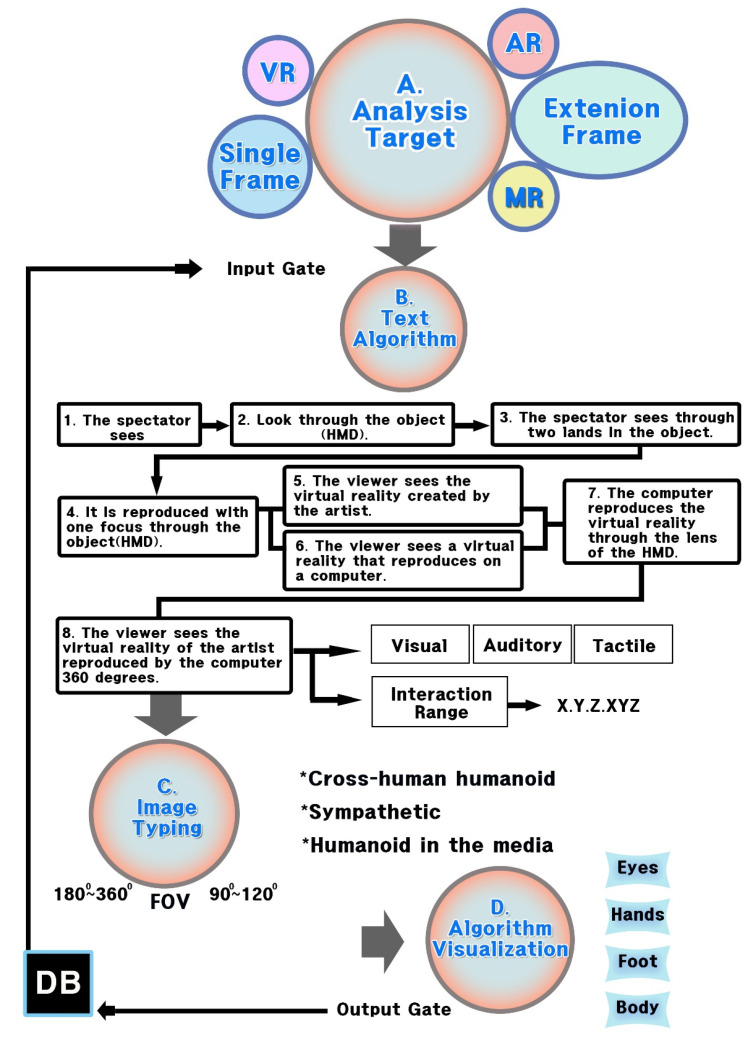
Flow chart of sensory content exercise ability detection algorithm.

Based on the database, the study demonstrated that VR e-sports had practical effects on exercise abilities and proposed a text algorithm to detect FOV and the scope of physical activities in the process of categorizing images and turning them into “notions of the real gaze”. Figure 6 shows the overall flow chart of an algorithm to detect exercise abilities in the proposed immersive content. As seen in this flow chart, the analysis framework to be applied to the experiment was divided into “B-1” of the device immersion methods (visual, auditory, and tactile) and “B-2” of the scope of interactions (X, Y, Z, and XYZ) in “B” of algorithms. In “C” of categorization, it was classified into “C-1” of FOV (90, 120, 180, and 360°) and “C-2” of frame immersion (cross-human humanoid, sympathetic, and humanoid in the media). In “D” of the “notion of the real gaze,” it was tested with “D-1” of users’ cognition methods (eyes, hands, feet, and bodies) and “D-2” of interaction methods (eyes, hands, feet, and bodies). These testing methods can help to predict the persistence and efficiency of exercise over time, based on result values.

## 4. Experimental Results and Discussion

### 4.1. B. Text Algorithm Analysis

#### 4.1.1. E-Sports Field

**Figure 7:**
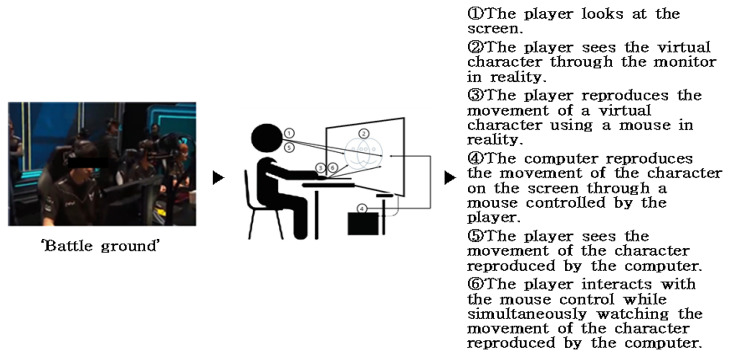
Example of experimental result of “text algorithm analysis” in the e-sports field.

Among the subjects of the experiment analysis, “Battleground” in the field of e-sports was analyzed with a text algorithm from the viewpoint of the user’s visual experience. The results are shown in Figure 7. As shown in the experimental results, the user establishes a communication relationship with the character displayed on the monitor screen and thinks that he and the character are the same. In addition, the sense of immersion is enhanced through mouse control. Due to the nature of the e-sports field, athletes immerse themselves in the characters moving on the monitor screen through a visual method. It is also connected to the tactile sense through mouse control. Additionally, the sound is transmitted through the headset as an auditory sensory experience. These results reveal the processes of how the user is immersed through the relationship with the device, and the result is to be subdivided into numerical values.

In the above results, we looked at the experimental results of “Battleground” in the field of e-sports, used as an experiment tool. Based on the results, the following case analysis was conducted. The result is shown in the following Table 8 text algorithm analysis result in e-sports field.

**Figure 8:**
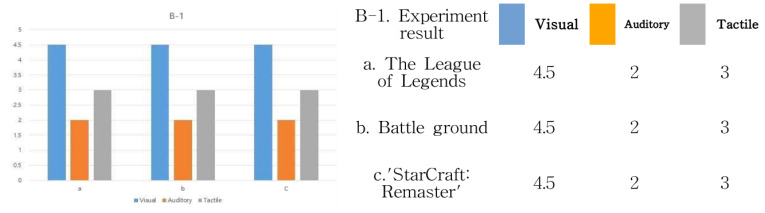
B-1 device immersion method test result.

Based on the above experiment results, let us analyze the results of (B-1) the device immersion method about “Battleground” in the e-sports field. The feeling of immersion is formed from the eyes of users ➀ and ➄ and the movement of the character in ➁. In addition, immersion is formed in the movements of the ➂ and ➅ mouse controls and the ➁ character. Therefore, if the device immersion method is subdivided into visual, auditory, and tactile senses, it can be seen that the visual parts of ➀, ➄, and ➁ and the tactile parts of ➂, ➅, and ➁ form a sense of immersion. In addition, the user’s headset creates an immersive feeling in the auditory part. In order to quantify these results, the degree of immersion in sight, hearing, and tactile sense based on ➄ is expressed as a number, and the results are shown in Figure 8. The rest of the cases were also tested in the same way.

**Figure 9:**
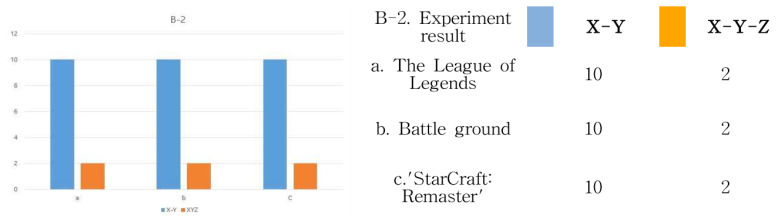
B-2 interaction range experiment results.

The analysis targets A. League of Legends (LoL), B. Battleground, and C. StarCraft: Remastered all experience through the same operating system, and the results are the same. In other words, vision is the largest, and next, a sense of immersion is formed through a controller using mouse manipulation. Next is B-2; let us look at the range of interactions in Figure 7. Basically, it is an experience method shown in “Battleground” in the field of e-sports. First of all, the user is seated. Therefore, since the monitor screen to be viewed is flat, the user’s gaze forms an interactive range following the movement of the character from the *x*-axis to the *y*-axis. In addition, it can be seen that the interaction of the mouse movement occurs only in a fixed position of the controller device. Therefore, based on 10, it can be seen that the range of interaction between the visual and mouse controller is mostly limited to the *X*-axis and *Y*-axis. In other words, it can be seen that the visual perception method on the monitor screen and the sense of immersion in mouse operation are flat, in that it is a 2D space. In addition, the same experimental results were found in League of Legends (LoL) and “StarCraft: Remastered” in the e-sports field. The results are shown in Figure 9. Other cases were also tested in the same way.

#### 4.1.2. VR E-Sports Field

**Figure 10:**
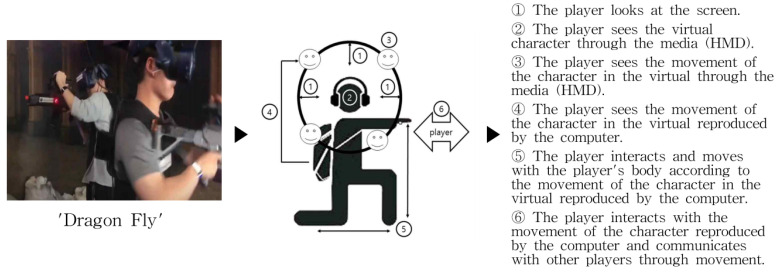
Example of experimental result of “text algorithm analysis” in the VR e-sports field.

The next experiment analyzes the “Dragon Fly” in the field of VR E-sports, among the analysis targets with a text algorithm from the viewpoint of the user’s visual experience. The results are shown in Figure 10 below. As can be seen from the experimental results, the user establishes a consensus between the character in the game and the user through the HMD and considers himself and the character as one. In addition, the sense of immersion is accelerated through game devices (weapons). Due to the nature of the VR e-sports field, players actually experience their physical movements in a 360-degree screen. The characters in the game are immersed in a complete virtual space through a visual method and connected with a tactile sense through a game device (weapon). The headset sound also accelerates your auditory immersion.

Let us analyze the final result, based on the experiment result of “Dragonfly” in the field of VR e-sports that was used as an experiment tool earlier. The following Table 9 shows the result of text algorithm analysis in the field of VR e-sports.

Based on the above experiment results, B-1, “save bits” in the VR e-sports field, analyzing the results of the device immersion method creates a sense of immersion in the visual parts of the user in ➀ and ➂ and the movement of the character in ➁ and ➂. In addition, immersion is formed through interaction between the movement of user ➄ and movement of character ➂. Therefore, subdividing into visual, auditory, and tactile senses, it can be seen that the sense of immersion is formed through the visual parts of ➀, ➂, and ➁ and the movement of the user’s body in ➄. In other words, the tactile sense of immersion is formed through the movement of the body. In addition, a sense of immersion is formed in the auditory part through the user’s headset.

In addition, the auditory part plays a large role in the game operation characteristics of Beat Saber, which has a strong musical element. Next, let us look at the analysis of “Dragon Fly”. Immersion is formed from the user’s gaze in ➀ and ➂ and the movement of the character in ➁. In addition, immersion is formed through interaction between the movement of user ➄ and movement of character ➂. In addition, with number ➅, immersion is also formed through communication with other users. Therefore, let us look at it by subdividing it into sight, hearing, and touch. It can be seen that immersion is formed through communication with the character through the visual parts of ➀, ➂, and ➁, the body movement in ➄, and the relationship with other users in ➅. In other words, the characteristic of “Dragon Fly” appeared that the tactile sense of immersion was formed through the movement of the body.

**Figure 11:**
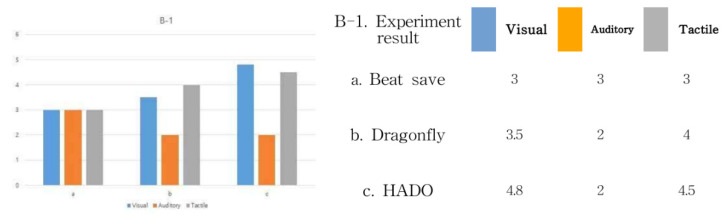
B-1 device immersion method test result.

In addition, immersion is being formed in the auditory part through the user’s headset. Finally, looking at the analysis of HADO, immersion is formed from the user’s perspective in ➀ and ➂ and the movement of the character in ➁. The characteristic part is that in HADO; the other user is seen along with the character image in ➂ and ➃. In other words, a virtual image and another user in reality are simultaneously visible through visual communication. In addition, in step ➄, communication through the movement of the character and the movement of the other user plays a large role. In addition, in step ➅, visual and tactile communication relationships are formed through communication with fellow teams, along with the movement of the virtual character and the other user. In order to quantify these results, the degree of immersion in visual, auditory, and tactile sensations, based on ➄, was expressed as a number. The results are shown in Figure 11.

Next, let us look at the “B-2” interaction range in Figure 11. First, in the experience method shown in Beat Saber, the monitor screen viewed by the user is completely immersive in a 360-degree three-dimensional space through the HMD, but the user is fixed in place to experience it. Therefore, the interaction range of the user’s gaze appears narrowly along the movement of the character from the *x*-axis to the *y*-axis. However, it can be seen that the user’s body moves up and down, left and right freely, and through interaction with the controller, there is a lot of movement of the user’s whole body, such as the hands and feet. Next, in the experience method shown in Dragonfly, the monitor screen viewed by the user is 360 degrees through the HMD, and a complete immersion is formed. However, unlike Beat Saber, users can move their body. Through communication with other users, the interaction of the body movement and device immersion is better than Beat Saber. However, in that the real space is blocked, due to the nature of the HMD, the inconvenience of moving appears as a disadvantage to the interaction.

**Figure 12:**
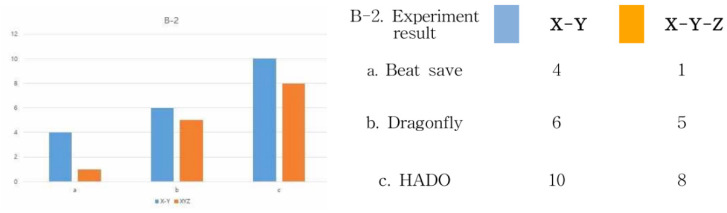
B-2 interaction range experiment results.

Finally, in HADO, there is no restriction on the movement of users, in that they can see both real and virtual images using the AR method. In addition, it can be seen that not only the interaction between the device and the user, but also the communication through the team and the competitive relationship with the other team expands the sense of immersion. Therefore, on the basis of 10, the range of visual interaction, device interaction, and user interaction could be analyzed by subdividing into *X*-axis, *Y*-axis and *X*-axis, *Y*-axis, *Z*-axis. In other words, in the interaction between the user and the device, there was a lot of body movement, due to the characteristics of the music and user interaction method of Beat Saber. However, when using the HMD, movement was formed only in a fixed position by blocking the eyes of the real space. In Dragonfly, interaction was additionally formed through communication between users. However, the movement of the body was limited, due to the blocking of reality by the HMD. However, in HADO, it can be seen that interaction is maximized through a game method that utilizes the relationship between users and the characteristic that real space and virtual images coexist. Therefore, the numerical values of the results are shown in Figure 12 below.

### 4.2. C. Typing Analysis

The result of categorization based on the features of the frame appearing in the field of e-sports and VR e-sports is shown in Table 10 below.

According to David Bodwell’s discussion, the frame was interpreted as a boundary concept of a domain called “a rectangular border that influences the degree to which the size of the situation in the screen is controlled and understood” [41]. This means the concept of a boundary between a formal and physical concept and a spatial category as an object in which a certain method or image is represented [42]. As can be seen from the above results, “League of Legends (LoL)”, “Battleground”, and “StarCraft: Remastered” in e-sports all appear in the same typology. The reason for this is basically that e-sports are played in front of a monitor. Therefore, players play games from a two-dimensional perspective of a monitor device and experience in reality through virtual images and visual interactions. According to Habert Zettle, the expansion of the aspect ratio as a horizontal aspect ratio from 4:3 to 16:9 is intended to satisfy human visual needs. It has been said that this may be because the human field of view is longer horizontally than vertically, and human life is made more horizontally than vertically [43]. Therefore, the sense of immersion may vary, depending on the size of the frame, but the method of operating in front of the monitor frame is the same. On the other hand, in the field of VR e-sports, “Beat Saber” and “Dragonfly” showed the same typification, and in “HADO”, different types of typification appeared. The reason is that “Beat Saber” and “Dragonfly” use HMD-based devices to completely block reality and experience a 360-degree virtual space in the visual. However, the disadvantage is that the user’s visual reality is blocked, and the body movement is not free. However, there is a difference in that “HADO” uses a device that uses AR. That is, by augmenting a virtual image in a real space and visually showing it, the reality and the virtual can be seen at the same time. In this respect, the advantage is that users can move freely and be more immersed in the game. As a result, the feeling of immersion increases when the monitor frame is enlarged or the viewing angle is visually expanded, rather than the feeling of immersion felt in the way of using a single monitor. Therefore, through these features, it can be seen that the user’s sense of immersion or interaction may vary, depending on which device is used.

**Figure 13:**
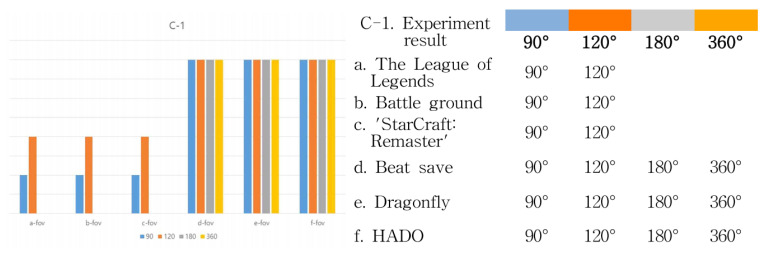
C-1 FOV range test result.

**Figure 14:**
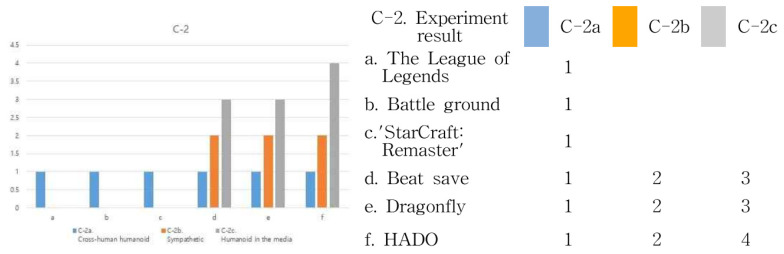
C-2 frame immersion test result.

Table 10 shows the “C-1” FOV range experiment and “C-2”. Let us look at the frame immersion experiment results. The FOV, or field of view, plays an important role in enhancing the user’s sense of immersion. Therefore, the larger the viewing angle, the higher the sense of immersion. This is also the case in “C-2”. In the frame immersion experiment, the feeling of immersion changed according to the interaction method between the device and the user. The way users interact with each other by touching them with their hands or entering a virtual space creates a greater sense of immersion than users who only visually look at the monitor. Therefore, through the experimental results, the sense of immersion between the analysis cases was quantified. Results can be seen in Figure 13 and Figure 14.

As can be seen from the results, perception of an object is from grasping the features of the external structure to recognize the morphological features of the proposed structure. Therefore, the expansion of the frame is the creation of a perceptual range in which the eye and the object to be seen are recognized as the processes of change appearing in the physical size, and the visual border as the visible limit area is determined. As shown in the above result, League of Legends, Battleground, and StarCraft: Remastered are formed from a 90 to a 120° viewing angle. However, in Beat Saber, Dragonfly, and HADO, the viewing angle was formed from 90 to 360°. Therefore, the result is that the sense of immersion is higher in d, e, and f. That is, it can be said that the user’s body movement is proportional to the visual range and the frame range of the device. Therefore, it can be said that the greater the range of physical activity of the user or the distance of visual movement, the greater the exercise effect. On the other hand, Beat Saber and Dragonfly visually block reality, and movement may be limited because the user can only move in a virtual space. However, HADO can see the real space and the virtual space at the same time, so it can be said that the user’s physical activity and immersion are the most among the six cases.

In Figure 14, C-2 frame immersion can also be seen in the experimental results. In step 1, “C-2a,” the cross-human humanoid method, the user only looks at the monitor device. In step 2, “C-2b,” the sympathetic method is a form in which the user interacts with the monitor device. Finally, in step 3, “C-2c,” the humanoid in the media method is a form in which the user interacts with the device through physical communication, so it can be said that the most immersive and physical activities are experienced. Looking at the results, in the first stage including League of Legends, Battleground, and StarCraft: Remastered, a cross-human humanoid method was shown. In Beat Saber, Dragonfly, and HADO, not only the 1st stage but also the 2nd and 3rd stages are visible. In particular, HADO shows the highest level of immersion and user activity among the three features.

### 4.3. D. Image of Gaze

Table 11 shows the gaze image of the target based on the features of the frame appearing in the field of e-sports and VR e-sports.

The process of visualizing a target’s gaze is a visual representation of a text algorithm in a form that graphically describes the visual system between the device and the user. Therefore, the simpler the image structure, the simpler the visual system, and the more complex the visual system, the more processes the images overlap. Looking at the results above, in League of Legends, Battle ground, and StarCraft: Remastered, the image of the gaze is the basic visual structure of the “gaze,” and the relationship between the user and the device are facing each other.

**Figure 15:**
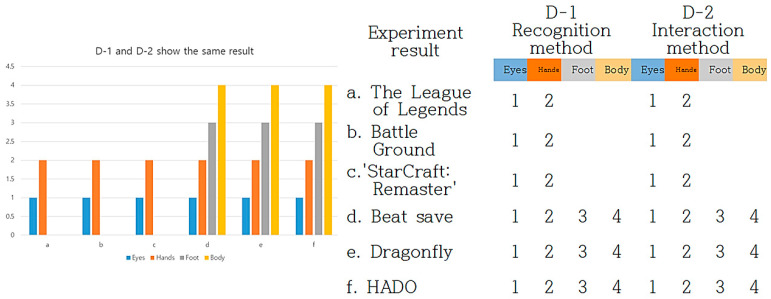
D-1. user recognition method and D-2. interaction method experimental results.

On the other hand, in Beat Saber and Dragonfly, the visual system forms a dual visual system in the virtual space, and it can be seen that the center of the user’s gaze exists in the virtual space. In addition, in HADO, it can be seen that not only the visual system, but also the user’s body is in the virtual space. Therefore, it can be seen that the user’s gaze and physical activity are immersed in the virtual space as it goes from a. to f. Based on this conclusion, the “D-1” user recognition method, and “D2,” let us look at the results of the interaction method. The results can be seen in Figure 15.

In Figure 15, “D-1” user recognition method and “D-2” interaction method can be found through the experimental results. In the cognitive method, apply a number from 1 to 4 in the order of eyes, hands, feet, and body. Considering that the eye is 1, it is judged that the degree of immersion has increased when both the eye and the hand are recognized at the same time. Therefore, 6 experimental cases are classified into “D-1” and “D-2,” and the result of the experiment increases from a to f, as shown above. The results of “D-1” and “D-2” are the same.

## 5. Conclusions

Following the outbreak of COVID-19, modern people have developed a need for a variety of health care content that they can enjoy safely at home. Their expectations and needs for immersive content are especially growing. A prominent need is found for killer content to satisfy both fun and health in today’s reality where people suffer a lack of physical activity. There have been diverse efforts to develop such content devices, but empirical analysis framework and tests of exercise effects are still in shortage and far from standardization. Thus, the present study set out to propose an analysis framework for e-sports equipped with both entertainment and sports elements and demonstrate the possibilities of VR e-sports.

The experiment results of the study show that analysis data of immersion and exercise abilities was generated between users and their devices in six research cases of e-sports and VR e-sports. Close relationships between users and their devices were found through text algorithm analysis under “B,” and the results were used to digitize “B-1” of device immersion methods and “B-2” of scope of interactions. Under “C” of categorization, the study measured and digitized “C-1” of FOV and “C-2” of frame immersion and obtained the independent result values of each device. Under “D” of “notion of the real gaze,” the study segmented “D-1” of users’ cognition methods and “D2” of interaction methods by the body part, identified physical characteristics according to the degree of immersion, and digitized the results. The final results were put in a diagram in Table 12.

These findings show that under “A” of objects of analysis, a variety of devices were used, based on game content in League of Legends, Battleground, StarCraft: Remastered, Beat Saber, Dragon Flight, and HADO. That is, while a, b, and c were managed in a way of visualizing planar frames on the monitor, d, e, and f used immersive content devices that were recently attracting attention and featured three-dimensional visual frames in 360 degrees. In particular, d and e offer excellent visual immersion by blocking a real space completely, but a lot of inconvenience is created, due to the reality that has been blocked. On the other hand, f can be managed in AR or MR to supplement this disadvantage, having great potential for utilization in the future. In the experiment results, the visual system between users and their devices was turned into an algorithm, based on texts under “B” of algorithm analysis. The results were used to digitize the results values of “B-1” of device immersion methods (visual, auditory, and tactile), according to the characteristics of each device. The result values indicate that immersion was great in visual parts overall. Moving from a to f, users increased their utilization of devices through their movements, based on their tactile part as much as their visual part, growing their physical activity level. Under “B-2” of scope of interactions, the study compared the X-, Y- and X-, Y-, and Z-axes in the scope of movements in physical activities, based on devices. Users used a device in a fixed position and managed their movements on the *X*, *Y*-axis in most cases in a, b, and c. However, in d, e, and f, they moved along the X-, Y-, and Z-axes by standing up to move around spaces and moving their arms and legs. In f, featuring a mixed reality between the real and virtual worlds, users were very active with their movements and had no limits in their scope of activities.

Under “C” of categorization, the study digitized “C-1” of FOV values in a process of turning text algorithms between users and their devices into images to figure them out more easily. Broader FOV meant higher immersion and expanded scope of physical activities. The result values show that users’ maximum FOV was within a scope of 90∼120° in a, b, and c, and that an FOV of 360° was basically created in d, e, and f. Allowing users to perceive reality freely, f especially maximized the scope of FOV. Under “C-2” of frame immersion, analysis was conducted in further segments of “C-2a,” cross-human humanoid, “C-2b,” sympathetic, and “C-2c,” humanoids in the media. Result values would vary according to whether there were physical contacts in relationships between users and their devices. Users would experience greater immersion when entering a virtual space and communicating with their devices by touching the screen, than when simply looking at the monitor screen. The outcome was in the form of “C-2a” in a, b, and c, in which users had no physical contact at all with the monitor screen offering virtual images. Both “C-2b” and “C-2c” were found in d, e, and f that were managed through users touching the screen and moving their bodies. Moving from d through e to f, users engaged in more active communication with their devices and experienced greater immersion and physical activity competence according to their movements and device operation methods.

Under “D” of “notion of the real gaze,” the study turned the characteristics of the experimentation process into visual algorithms. Any devices can be visualized through “notion of the real gaze” in the visualization process of information. The result values were used to digitize “D-1” of users’ cognition methods. They showed in which body parts the users had the greatest immersion in their devices through their eyes, hands, feet, and bodies, and in which parts they exhibited the highest activity. In a, b, and c, they were mostly managed through eyes and hands with physical movements immensely focused on visual parts. However, in d, e, and f, users were able to move their bodies through the movement of their feet, as well as their eyes and hands. In f, their body activity performed with the greatest excellence and induced decisive immersion in users. Moving from a to f, users recorded increasingly higher physical utilization and had immersion through the movement of their entire bodies, instead of physical parts. In “D2” of interaction methods, a, b, and c enabled communication through eyes and hands, whereas d, e, and f allowed users to interact with their devices through the movement of their feet and bodies, as well as their eyes and hands.

As a result, the results of using the analysis tool among A. analysis targets a. League of Legends, b. Battle Ground, c. StarCraft: Remastered, d. Beat Save, e. Dragon Fly, and f. HADO. It was found that the most effective example of the value was f. HADO. Based on these results, the form of realistic health care to be produced in the future requires a structure that has high frame scalability and can compete or cooperate with others online. In addition, it can be conveniently used at home, and exercise prevention and rehabilitation effect through safety, space expandability, and excellent immersion are considered to be great.

The present study mentioned a need for immersive content and research in the field of health care during the COVID-19 era, analyzing various immersive content in related research and identified an analysis framework to measure exercise abilities. The findings are presented in Table 13. The study put in various devices, conducted an experiment with an analysis framework, and tested their effects through digitization in the process of A-B-C-D.

The present study offered a set of criteria to analyze and understand immersive content that was further diversified and advanced. It is difficult to evaluate various devices with a simple technical approach. Because the human body connectivity of recent immersive devices is very high. Therefore, evaluation criteria suitable for the new digital visual system and criteria for verifying various devices are presented in this research paper. Until now, there is no clear health care product through VR technology in the health care field.

However, we are living in an era in which modern life patterns are changing and global epidemics appear. Recently, research on safe immersive devices to be used and revived in anticipation of a new virtual space called metaverse has already begun. Therefore, it is expected that the psychological and physical evaluation criteria for future health care products will be newly presented through this study. It is expanded from the current 2D-based visual system to a 360-degree 3D-based visual system. That is, an extended evaluation standard from the UI/UX perspective between humans and media is formed. In addition, various health care products using VR and AR formats can be produced. Finally, even if a pandemic such as COVID-19 recurs, it is expected that anyone from the elderly to children can enjoy exercise and prepare for prevention and rehabilitation through a safe, convenient, and fun way at home. In addition, I hope that this research paper will serve as a guide in producing products with both fun and exercise effects in the process of producing home health care products in the future. The study also raised a need for additional researches with many practical experiments and clearer and more effective researches, based on collaboration with medical and health care professionals. Everyone hopes that this will be the last virus crisis. If another one happens in the future, they will hopefully come up with wiser measures than the current situation and convey messages of overcoming and hope, rather than frustration and fear to the lives of modern people.

## Figures and Tables

**Table 1:** Image tangible and visual illustration of realistic content cases.

**Single Frame**	**Extension Frame**	**VR**	**AR**	**MR**	**Hologram**
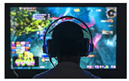	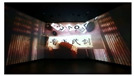	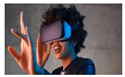	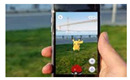	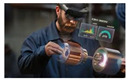	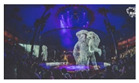
**Image Typing**					
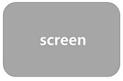	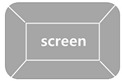	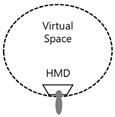	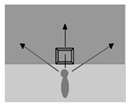	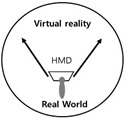	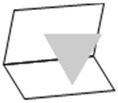
**FOV**					
90°’	180°~270°	360°	360°	360°	360°
**Algorithm Visualization**					
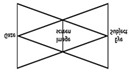	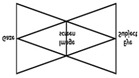	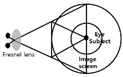	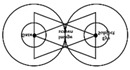	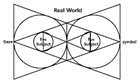	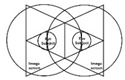

**Table 2:** Three cases of interactive type.

(1) Hive “Iris” 2012	(2) Air-Screen Interactive 7.5 m^2^ 2013	(3) Rodrigo Carvalho “Break Down” 2014
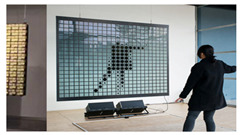	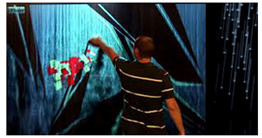	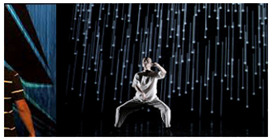

**Table 3:** Interactive visual plate.

	Cross-Human Humanoid	Sympathetic	Humanoid in the Media
Interactive Case	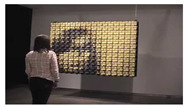	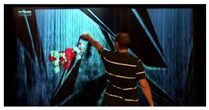	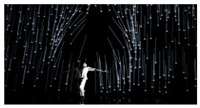
Interactive type	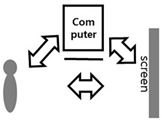	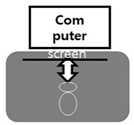	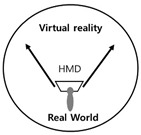
Visual plate	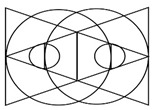	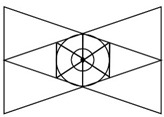	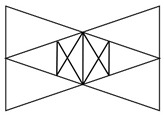

**Table 4:** Analysis target.

**E-sports**	League of Legends	Battleground	StarCraft: Remastered
	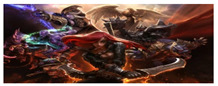	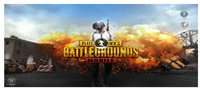	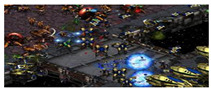
**VR e-sports**	VR Beat Saber	VR Dragon Flight	VR HADO
	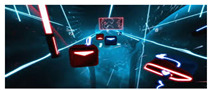	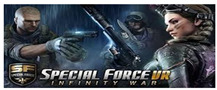	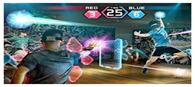

**Table 5:** Immersive device case.

Single Frame	Extension Frame	VR	AR	MR
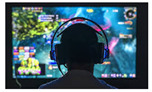	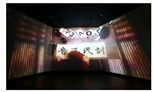	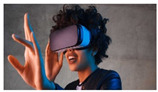	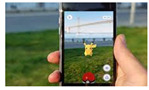	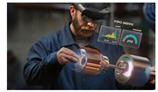

**Table 6:** Analysis tools and standards for experimentation.

Analysis Tool	A. Analysis Object	B. Algorithm				C. Typification				D. Diagram of Gaze			
Analysis standard	A. Immersive Device	**B-1. Device immersion method**				**C-1. FOV**				**D-1. User recognition method**			
		Visual		Auditory	Tactile	90	120	180	360	Eyes	Hands	Foot	Body
		**B-2. Interaction range**				**C-2. Frame immersion**				**D-2. Interaction method**			
		x	Y	z	xyz	C-2a. Cross-human humanoid				Eyes	Hands	Foot	Body
						C-2b. Sympathetic							
						C-2c. Humanoid in the media							

**Table 7:** Numericalization method for measuring exercise effect.

Numericalization Tool	Digitization Method	Numericalization Range
**B-1. Device immersion method**	Visual	**Depending on the level of physical use** **1~5**
	Auditory	
	Tactile	
**B-2. Interaction range**	X-Y	**Depending on the scope of the interaction** **1~10**
	X-Y-Z	
**C-1. FOV**	90°	**According to the line of sight** **90°~360°**
	120°	
	180°	
	360°	
**C-2. Frame immersion**	C-2a. Cross-human humanoid	**Steps 1 to 3 depending on the device and the degree of immersion of the human body**
	C-2b. Sympathetic	
	C-2c. Humanoid in the media	
**D-1. User recognition method**	1. Eyes	**According to the range of body use** **1~4**
	2. Hands	
	3. Feet	
	4. Body	
**D-2. Interaction method**	1. Eyes	
	2. Hands	
	3. Feet	
	4. Body	

**Table 8:** Text algorithm analysis result in e-sports field.

Analysis Target E-Sports	Text Algorithm
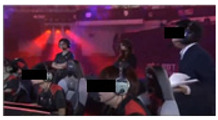 a. League of Legends (LoL)	➀. The player looks at the screen.
	➁. The player sees the virtual character through the monitor in reality.
	➂. The player reproduces the movement of a virtual character using a mouse in reality.
	➃. The computer reproduces the movement of the character on the screen through a mouse controlled by the player.
	➄. The player sees the movement of the character reproduced by the computer.
	➅. The player interacts with his mouse control, while watching the movement of the character reproduced by the computer.
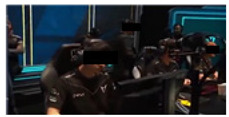 b. Battleground	➀. The player looks at the screen.
	➁. The player sees the virtual character through the monitor in reality.
	➂. The player reproduces the movement of a virtual character using a mouse in reality.
	➃. The computer reproduces the movement of the character on the screen through a mouse controlled by the player.
	➄. The player sees the movement of the character reproduced by the computer.
	➅. The player interacts with the mouse control, while simultaneously watching the movement of the character reproduced by the computer.
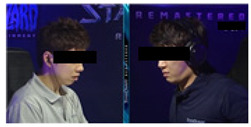 c. StarCraft: Remastered	➀. The player looks at the screen.
	➁. The player sees the virtual character through the monitor in reality.
	➂. The player reproduces the movement of a virtual character using a mouse in reality.
	➃. The computer reproduces the movement of the character on the screen through a mouse controlled by the player.
	➄. The player sees the movement of the character reproduced by the computer.
	➅. The player interacts with the mouse control, while simultaneously watching the movement of the character reproduced by the computer.

**Table 9:** VR e-sports text algorithm analysis result.

Analysis Target VR E-Sports	Text Algorithm
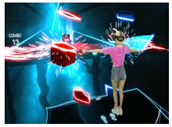 a. Beat Saber	➀. The player looks at the screen.
	➁. The player sees the virtual character through the media (HMD).
	➂. The player sees the movement of the character in the virtual through the media (HMD).
	➃. The player sees the movement of the character in the virtual reproduced by the computer.
	➄. The player interacts and moves with the player’s body according to the movement of the character in the virtual reproduced by the computer.
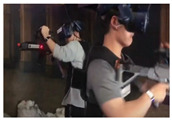 b. Dragonfly	➀. The player looks at the screen.
	➁. The player sees the virtual character through the media (HMD).
	➂. The player sees the movement of the character in the virtual through the media (HMD).
	➃. The player sees the movement of the character in the virtual reproduced by the computer.
	➄. The player interacts and moves with the player’s body according to the movement of the character in the virtual reproduced by the computer.
	➅. The player interacts with the movement of the character reproduced by the computer and communicates with other players through movement.
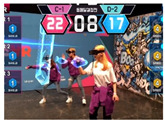 c. HADO	➀. The player looks at the screen.
	➁. The player sees the virtual image (character) and reality at the same time through the media (HMD).
	➂. The player sees the motion of the virtual image (character) reproduced by the computer and the opponent in reality at the same time.
	➃. The movement of the player is recognized by the computer and reproduced as an image (character) in the virtual. It also shows the opponent’s movement in reality at the same time.
	➄. The player interacts with the player’s body according to the motion of the virtual image (character) reproduced by the computer and moves with the opponent’s movement in reality.
	➅. Athletes interact with fellow (team) players, while interacting with the movement of images (characters) reproduced by a computer. Communicate through movement.

**Table 10:** Visualization of the analysis target frame.

Analysis Target	Frame Format		
E-sport	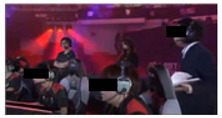	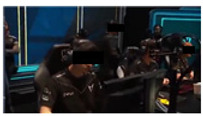	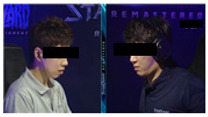
	League of Legends (LoL)	Battleground	StarCraft: Remastered
	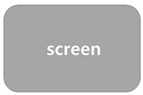	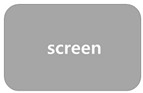	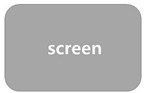
VR e-sport	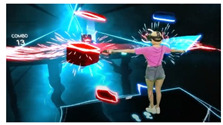	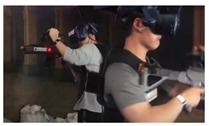	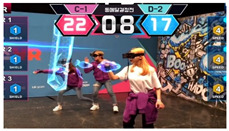
	Beat Saber	Dragonfly	HADO
	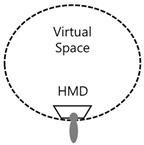	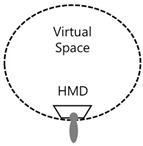	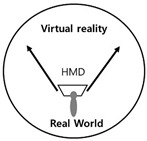

**Table 11:** Image of the target’s gaze.

a. League of Legends	b. Battleground	c. StarCraft: Remastered	d. Beat Saber	e. Dragonfly	f. HADO
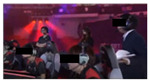	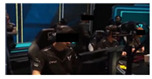	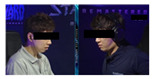	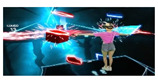	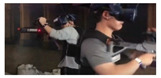	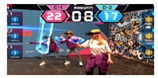
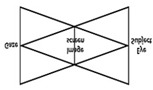	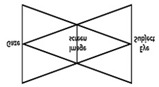	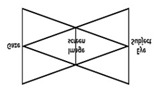	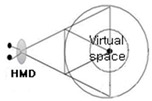	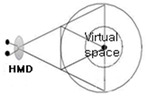	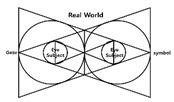

**Table 12:** Final experiment result.

A. Analysis Target			a. League of Legends	b. Battleground	c. StarCraft: Remastered	d. Beat Saber	e. Dragonfly	f. HADO
			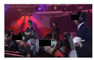	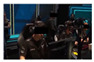	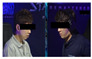	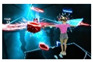	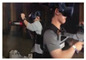	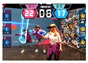
B. Text algorithm analysis	B-1. Device immersion	Visual	4.5	4.5	4.5	3	3.5	4.8
		Auditory	2	2	2	3	2	2
		Tactile	3	3	3	3	4	4.5
	B-2. Interaction range	X-Y	10	10	10	4	6	10
		X-Y-Z	2	2	2	1	5	8
C. Typification	C-1. FOV	90°	90°	90°	90°	90°	90°	90°
		120°	120°	120°	120°	120°	120°	120°
		180°	-	-	-	180°	180°	180°
		360°	-	-	-	360°	360°	360°
	C-2. Frame immersion	C-2a. Cross-human humanoid	1	1	1	1	1	1
		C-2b. Sympathetic	-	-	-	2	2	2
		C-2c. Humanoid in the media	-	-	-	3	3	3
D. Diagram of gaze	D-1. User recognition method	1. Eyes	1	1	1	1	1	1
		2. Hands	2	2	2	2	2	2
		3. Feet	-	-	-	3	3	3
		4. Body	-	-	-	4	4	4
	D-2. Interaction method	1. Eyes	1	1	1	1	1	1
		2. Hands	2	2	2	2	2	2
		3. Feet	-	-	-	3	3	3
		4. Body	-	-	-	4	4	4

**Table 13:** Realistic content exercise effect verification tool.

Analysis Method	Analysis Tool	Numericalization Process
A. Analysis target		
B. Text algorithm analysis	B-1. Device immersion method	Visual
		Auditory
		Tactile
	B-2. Interaction range	X-Y
		X-Y-Z
C. Typification	C-1. FOV	90°
		120°
		180°
		360°
	C-2. Frame immersion	C-2a. Cross-human humanoid
		C-2b. Sympathetic
		C-2c. Humanoids in the media
D. Image of gaze	D-1. User recognition method	1. Eyes
		2. Hands
		3. Feet
		4. Body
	D-2. Interaction method	1. Eyes
		2. Hands
		3. Feet
		4. Body

## Data Availability

Not applicable.
